# mHealth Intervention to Improve Diabetes Risk Behaviors in India: A Prospective, Parallel Group Cohort Study

**DOI:** 10.2196/jmir.5712

**Published:** 2016-08-05

**Authors:** Angela Pfammatter, Bonnie Spring, Nalini Saligram, Raj Davé, Arun Gowda, Linelle Blais, Monika Arora, Harish Ranjani, Om Ganda, Donald Hedeker, Sethu Reddy, Sandhya Ramalingam

**Affiliations:** ^1^ Northwestern University Feinberg School of Medicine Chicago, IL United States; ^2^ Arogya World Naperville, IL United States; ^3^ Health ARC Pennington, NJ United States; ^4^ Focus Scientific Research Center Bengaluru India; ^5^ Emory Centers for Training and Technical Assistance Behavioral Sciences and Health Education Emory University Atlanta, GA United States; ^6^ Public Health Foundation of India Haryana India; ^7^ Madras Diabetes Research Foundation Chennai India; ^8^ Joslin Diabetes Center Boston, MA United States; ^9^ University of Chicago Chicago, IL United States

**Keywords:** mHealth, diabetes, health promotion

## Abstract

**Background:**

In low/middle income countries like India, diabetes is prevalent and health care access limited. Most adults have a mobile phone, creating potential for mHealth interventions to improve public health. To examine the feasibility and initial evidence of effectiveness of mDiabetes, a text messaging program to improve diabetes risk behaviors, a global nonprofit organization (Arogya World) implemented mDiabetes among one million Indian adults.

**Objective:**

A prospective, parallel cohort design was applied to examine whether mDiabetes improved fruit, vegetable, and fat intakes and exercise.

**Methods:**

Intervention participants were randomly selected from the one million Nokia subscribers who elected to opt in to mDiabetes. Control group participants were randomly selected from non-Nokia mobile phone subscribers. mDiabetes participants received 56 text messages in their choice of 12 languages over 6 months; control participants received no contact. Messages were designed to motivate improvement in diabetes risk behaviors and increase awareness about the causes and complications of diabetes. Participant health behaviors (exercise and fruit, vegetable, and fat intake) were assessed between 2012 and 2013 via telephone surveys by blinded assessors at baseline and 6 months later. Data were cleaned and analyzed in 2014 and 2015.

**Results:**

982 participants in the intervention group and 943 in the control group consented to take the phone survey at baselne. At the end of the 6-month period, 611 (62.22%) in the intervention and 632 (67.02%) in the control group completed the follow-up telephone survey. Participants receiving texts demonstrated greater improvement in a health behavior composite score over 6 months, compared with those who received no messages F(1, 1238) = 30.181, *P*<.001, 95% CI, 0.251-0.531. Fewer intervention participants demonstrated health behavior decline compared with controls. Improved fruit, vegetable, and fat consumption (*P*<.01) but not exercise were observed in those receiving messages, as compared with controls.

**Conclusions:**

A text messaging intervention was feasible and showed initial evidence of effectiveness in improving diabetes-related health behaviors, demonstrating the potential to facilitate population-level behavior change in a low/middle income country.

**Trial Registration:**

Australian New Zealand Clinical Trials Registry (ACTRN): 12615000423516; https://www.anzctr.org.au/Trial/Registration/TrialReview.aspx?id=367946&isReview=true (Archived by WebCite at http://www.webcitation.org/6j5ptaJgF)

## Introduction

Diabetes is estimated to affect 387 million globally, with disease prevalence expected to increase to 592 million by the year 2035 [1]. Approximately 80% of people with diabetes live in low- and middle-income countries (LMICs) creating a pressing need for prevention and treatment efforts focused on these regions. With 3.6 billion mobile subscriptions worldwide in 2014, mobile phones hold great potential as an intervention delivery channel [2]. Mobile phone uptake is high, even in parts of the globe that lack basic electricity and sanitation infrastructure. Especially since the mobile phone is carried by the user throughout much of the day, mobile health intervention holds the potential to reach and help remote target populations cost-effectively [2,3].

Text messaging interventions could offer a particularly useful health promotion intervention delivery channel for LMICs. Texts can be delivered to the most basic mobile phones and do not require consistent connectivity or Internet capability. Hence, messaging can be delivered inexpensively and automatically to widely geographically dispersed people from different socioeconomic strata [4]. Recent systematic reviews of text messaging health promotion interventions indicate that text messaging has not only proved most efficacious for smoking cessation but also shows some promise for improving other health behaviors, yielding small-to-medium effects comparable with those of print- or computer-delivered interventions [5,6]. However, the vast majority of studies to date have been conducted in high-income countries and in a single language [7]. Since LMICs are disproportionally affected by diabetes as well as underresourced compared with higher income countries, research specifically examining text messaging intervention as part of a public health initiative in LMICs is needed.

India is illustrative of other LMICs, in having a high prevalence of diabetes, poor health care access, and yet a high penetration of mobile device use. Despite its relatively low rates of overweight and obesity, more than 62 million of India’s 1.2 billion residents are diagnosed with diabetes [8,9]. The size and heterogeneity of India’s population, limited access to health services, and scarcity of quality-controlled clinical laboratory facilities create barriers to intervening preventively on diabetes. Access challenges are particularly acute in India’s rural areas, where almost 70% of the population resides [10]. Growing evidence indicates that Indians have heightened genetic risk and a lowered disease threshold in response to diabetes risk factors including age, obesity, abdominal adiposity, and high body fat percentage, resulting in increased risk of diabetes at younger age and lower body mass than other ethnic groups [8]. The elevated and increasing risk of diabetes in the Indian population creates an urgent need for effective interventions that can be scalable to all regions. With its large number of mobile phone subscribers (900 million) [11], India offers a test bed to examine whether an mHealth intervention has the potential to reduce diabetes risk in a LMIC population.

To date, few chronic disease prevention interventions have been tested in the Indian population. Two studies using in-person or telephone counseling showed feasibility, acceptability, and preliminary evidence of efficacious screening and treatment for prediabetic and diabetic patients in rural India. However, these interventions used either individual telephone counseling or a fully equipped mobile van [12,13], requiring extensive personnel and equipment resources that preclude scalability and national implementation in an LMIC. To date, the sole test of a diabetes-related text messaging intervention in India was conducted in men with impaired glucose tolerance, limiting generalizability to the context of prevention in those at risk, rather than population-level prevention [14]. To slow the epidemic of diabetes in India, scalable prevention interventions are needed that can address cultural, geographical, and language barriers across the entire population.

mDiabetes was a text message, public health program developed to address awareness of diabetes and the corresponding risk behaviors. The program was planned to be disseminated through Nokia’s mobile platform. The investigators leveraged an opportunity to employ a pre-post evaluation of effectiveness of the messages disseminated. Thus, this study aimed to evaluate whether this 6-month text messaging intervention alone, unaccompanied by costly, burdensome in-person visits or telephone coaching, was acceptable to end users and could improve behavioral risk factors for diabetes in all segments of the Indian population. We hypothesized that those receiving the text messaging intervention, as compared with an untreated comparison group, would show positive changes in 4 health behaviors that lessen diabetes risk: engagement in exercise, avoidance of fat foods, fruit intake of 2 servings a day or more, and vegetable intake of 2 servings a day or more. Furthermore, we explored whether the intervention increased the prevalence of those able to change more than a single health behavior, an important outcome since risk behaviors cluster, and diabetes prevention requires engaging in multiple healthy behaviors [15,16].

## Methods

### Study Design and Participants

Text messages for the mDiabetes program were developed by Emory University and reviewed by a Behavior Change Task Force assembled as part of a 2011 Clinton Global Initiative Commitment by Arogya World. The 56 messages were designed to motivate improvement in diabetes risk behaviors and increase awareness about the causes and complications of diabetes. Based on feedback from Indian consumers, messages were culturally tailored to be more acceptable and actionable by the population. In 2012, Nokia held a 22% marketshare in India, [17] which enabled them to invite one million individuals from all over India to opt in to receive health messages; enrollment was closed once that number was reached. Texts (see Multimedia Appendix 1) were available in one of 12 languages based on participant preference. Texts arrived in a predetermined order and frequency (twice a week) to a dedicated inbox on the Nokia phone. A comparison group was drawn from a database that included all mobile phone users in India, after excluding subscribers to Nokia service.

Ipsos, a global market research company, implemented the evaluation by randomly selecting samples to be interviewed from the Nokia and non-Nokia cohorts and conducting the phone surveys in multiple languages. Intervention participants and controls were recruited in India for a 6-month prospective study. The intervention group of 982 was randomly selected from the one million Nokia phone customers who opted in to receive mDiabetes messages. The control group (n=943) was randomly selected from the database including all non-Nokia mobile phone users in India. The sole eligibility criteria were that participants in both groups be adults aged 18 years and older. The research protocol was approved by an independent ethics review committee of the Centre for Chronic Disease Control of India, New Delhi. The protocol was registered in the Australian New Zealand Clinical Trials Registry, #ACTRN12615000423516.

### Procedures

Between November and December of 2012, Ipsos staff assessed baseline levels of behaviors by interviewing all study participants by mobile telephone in the participant’s choice of language. A similar follow-up interview was repeated 6 months later in mid 2013. Interviews were scripted, conducted by personnel who were kept blind to the interviewee’s treatment assignment, and lasted approximately 20 minutes. The baseline and 6-month interviews comprised 19 questions asking participants to self-report demographic information, including residential location, age, and health behaviors. To assess physical activity, participants were asked, “Do you exercise currently?” with response options “yes” or “no.” Number of fruit and of vegetable servings was assessed with response options of “0 to 1 servings, 2-3 servings, or 4 or more servings.” High fat food intake was assessed with the question, “Do you consistently avoid eating high fat food/fried food such as samosas, vadai, bajji, bondas, etc.?” with response options “ yes” or “no.” Diabetes preventive behaviors were coded as: endorsing exercise, endorsing avoidance of fatty food, endorsing consuming 2 or more servings of fruit, endorsing consuming 2 or more servings of vegetables.

Over 6 months, participants in the active intervention condition were sent 56 unique messages related to diabetes: one message per day for the first 6 days, then 2 messages per week. Participants in the control group received no study contact until the end of the 6-month period.

### Statistical Analysis

Data checking, cleaning, and analyses were conducted in 2014 and 2015. The study was designed to detect a 10% difference between groups on change in a composite health behavior score with 80% power and a 2-sided significance level of α=0.05. Power analysis produced a sample size estimate of 384 for each group. Assuming that rural versus urban location would be a meaningful covariate, the objective was to recruit equal numbers in each area. Therefore, with an estimated 15% attrition, target recruitment was 450 participants for intervention and control, stratified by geographic region, resulting in a total sample size of at least 1800.

The match between each individual’s postintervention and preintervention survey was confirmed on the basis of phone number and demographic data. Differences between the intervention and control groups in baseline measures and retention were tested by chi-square. All available surveys were included in the baseline analyses, but only matched pairs of surveys were included in the longitudinal analysis. To compare the lifestyle behavior changes over time, a composite healthy behavior change score was constructed. Each instance of pre/postbehavior change from unhealthy status to healthy status was assigned a numeric score of 1; no change was assigned a score of 0; change from healthy to unhealthy was assigned a score of −1. The scores for each of the 4 behaviors were then summed, generating a composite change score that ranged from 4 (when all 4 behaviors moved from unhealthy status in the prephase to healthy status after treatment) to −4 (when all 4 behaviors changed from healthy to unhealthy). Healthful behavior change was operationalized by the composite healthy lifestyle improvement score [18].

Normality of the distribution of the composite score was evaluated before conducting the primary analysis comparing the 2 treatment groups on healthful behavior change. An analysis of covariance was conducted with baseline level of behavior, gender, and urban/rural location included as covariates. Secondary analyses using logistic regression with the same covariates compared the groups on the presence of each of the 4 preventive behaviors at postintervention. All analyses were performed using in IBM SPSS Statistics (version 22) [19].

## Results

During the recruitment phase of the study, 982 participants in the intervention group and 943 in the control group consented to take the phone survey (Table 1). A majority of the sample were male (88.52%), lived in an urban location (68.78%), and resided in the North of India (67.06%). At both baseline and 6-month follow-up, the intervention group had fewer males (*P*<.001) and fewer individuals living in urban locations (*P*<.001) than the control group. At the end of the 6-month period, 611 (62.22%) in the intervention and 632 (67.02%) in the control group completed the follow-up telephone survey (*P*=.028).

**Table 1:** Participant baseline characteristics.

	Overall; n	Control; n	Experimental; n	*P* value
Baseline	1925	943 (48.99%)	982 (51.01%)	
Male	1704 (88.52%)	881 (93.43%)	823 (83.81%)	<.001^a^
Urban	1324 (68.78%)	867 (91.94%)	457 (46.54%)	<.001^a^
North India	1291 (67.06%)	653 (69.25%)	638 (64.97%)	.047^a^
Mean age (SD)	32.2 (10.6)	32.83 (9.39)	31.66 (11.64)	.016^a^
Consumes fruit	46 (2.39%)	31 (3.29%)	15 (1.53%)	.033^a^
Consumes vegetables	75 (3.90%)	53 (5.62%)	22 (2.24%)	<.001^a^
Consumes fat	569 (29.56%)	267 (28.31%)	302 (30.75%)	.241
Exercises	1094 (56.83%)	601 (63.73%)	493 (50.20%)	<.001^a^

^a^It indicates statistical significance (*P*<.05).

**Figure 1:**
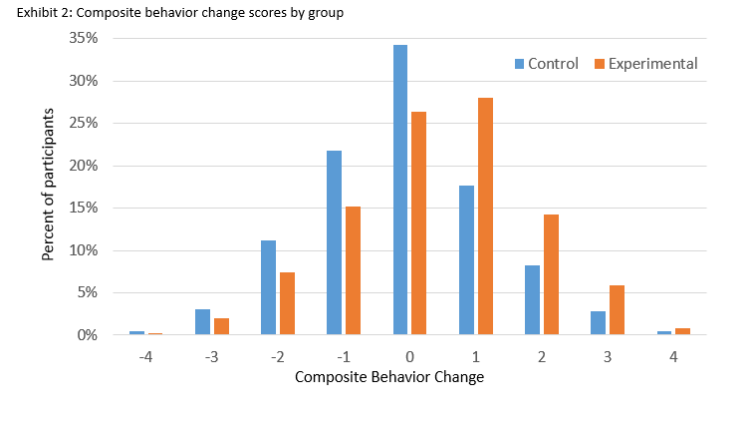
Composite behavior change scores by group.

After controlling for covariates (baseline behavior, gender, urban/rural location), the treatment groups differed significantly in their composite healthy change scores F(1, 1238) = 30.181, *P*<.001, Bonferroni adjusted 95% CI (0.251-0.531), such that participants in the intervention group reported greater improvement in their aggregated diabetes risk behaviors over time than participants in the control group. Figure 1, demonstrating the distribution of change scores for each group, indicates that 36.55% of participants in the control condition reported a decline in their number of healthy behaviors, as compared with 24.71% of those in the intervention group.

**Figure 2:**
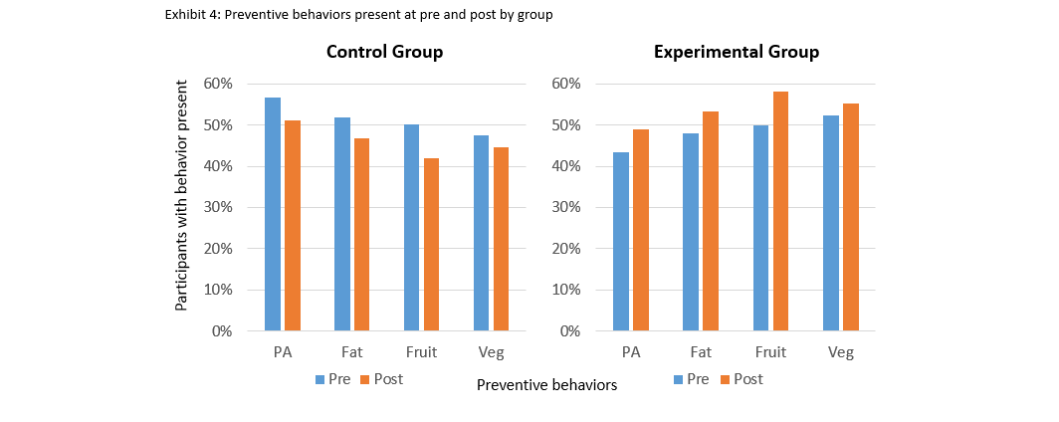
Preventive behaviors present at pre and postintervention by group.

Analyses shown in Table 2 revealed that, as compared with controls, more participants in the experimental group improved their fruit and vegetable intake and reduced their fat intake after intervention. No differential change in exercise was observed between the groups (Figure 2). In addition, 128 (20.95%) of the intervention group compared with 73 (11.55%) of the control group improved 2 or more health behaviors.

**Table 2:** Logistic regression of behaviors at the end of intervention by group with covariates.

Health Behavior	Predictor	*β*	SE *β*	Wald’s χ²	dƒ	p	e^β^
Fruit Consumption (n=1243)	Constant	−0.836	0.098	72.611	1	<.001^a^	0.434
	Baseline	0.543	0.122	19.797	1	<.001^a^	1.721
	Location	0.230	0.141	2.657	1	.103	1.259	
	Gender	−0.132	0.199	0.441	1	.507	0.876
	Group	0.549	0.133	17.023	1	<.001^a^	1.731
	Test			χ²	dƒ	P	
	Overall model evaluation			51.919	4	<.001^a^	
							
Vegetable Consumption (n=1243)	Constant	−0.357	0.110	10.452	1	<.001^a^	0.700
	Baseline	0.984	0.124	62.628	1	<.001^a^	2.674	
	Location	0.135	0.153	0.774	1	.379	1.144
	Gender	0.217	0.220	0.972	1	.324	1.242
	Group	0.561	0.140	16.025	1	<.001^a^	1.753
	Test			χ²	dƒ	P	
	Overall model evaluation			98.424	4	<.001^a^	
							
Fat Consumption (n=1243)	Constant	0.465	0.134	12.055	1	.001^a^	1.593	
	Baseline	0.463	0.144	10.332	1	.001^a^	1.589
	Location	0.446	0.179	6.215	1	.013^a^	1.562
	Gender	0.462	0.271	2.902	1	.088	1.587
	Group	0.510	0.156	10.739	1	.001^a^	1.665
	Test			χ²	dƒ	P	
	Overall model evaluation			46.373	4	<.001^a^	
							
Physical activity (n=1243)	Constant	−0.163	0.113	2.094	1	.148	0.849
	Baseline	0.963	0.121	63.347	1	<.001^a^	2.620
	Location	0.009	0.145	0.004	1	.951	1.009
	Gender	0.048	0.203	0.055	1	.815	1.049
	Group	0.094	0.137	0.469	1	.494	1.098
	Test			χ²	dƒ	P		
	Overall model evaluation			65.295	4	<.001^a^	

^a^ indicates statistical significance (*P*<.05).

## Discussion

### Principal Findings

This study demonstrated the feasibility and initial evidence of effectiveness of a diabetes prevention text messaging intervention in 12 languages delivered to a sample of nearly 1000 Indian adults, drawn from both rural and urban regions. As compared with those who were not sent diabetes-related messages, those receiving text messages designed to enhance awareness of diabetes risk factors displayed greater improvement in diabetes preventive behaviors over a 6-month period. In addition, the text messaging intervention prevented health behaviors from deteriorating. Importantly, the minimalist and scalable text messaging intervention improved more than one risk behavior simultaneously, an important feature since multiple co-occurring risk behaviors are implicated in the development of diabetes and other chronic diseases. Fat and fruit and vegetable intakes all improved at 6 months in the group receiving messaging, as compared with controls after controlling for baseline differences between the groups. Even though the group receiving text messages reported a small increase in exercise relative to controls, the difference was not significant. Consistent with findings from other studies, a more robust intervention may be needed to produce improvement in physical activity [18]. Overall, results support the feasibility, acceptability, and preliminary effectiveness of a low-cost, low-burden text messaging intervention to prevent degradation of health behaviors over time and to promote the acquisition of diabetes preventive behaviors.

Diabetes imposes a devastating socioeconomic burden globally, especially in the developing world. Although effective diabetes preventive interventions exist, including those evaluated in the Diabetes Prevention Program, the Finnish Diabetes Prevention Study, the Malmo Study of Sweden, and the Da Qinq IGT and Diabetes Study of China, most successful treatments are very burdensome and costly [20-23]. An urgent need exists to develop effective, scalable interventions that are adaptable for different cultures at low per-patient cost. As mobile phone use continues to increase, the use of mHealth interventions to address public health problems grows increasingly feasible and attractive [23]. Results of this study adds to the emerging evidence that population level healthy lifestyle change may be attainable through low-cost mobile health interventions [24].

### Limitations

This study had several limitations that should be considered when interpreting the results. First, the absence of random assignment to treatment groups resulted in some imbalances in the baseline characteristics of the groups. We addressed these by controlling for covariates in the analyses; however, it is possible that inherent differences between the intervention and control populations remained. Second, the selection of participants may have been biased toward those having greater than average interest in health, given that all participants agreed to answer questions about their health. Measurement error and social desirability bias may have been operative since participants’ behaviors were self-reported rather than objectively measured. Although these selection biases are of concern, they apply similarly and nondifferentially to participants in both groups. This study was constrained by time and cost of asking multiple questions in multiple languages and as such was unable to use longer, more rigorous assessments of diet quality. Thus, to assess initial feasibility and potential for public health benefit, brief questions were used. Another measurement challenge that affected both groups was that, to avoid appearing rude or insulting, interviewers inferred the participant’s gender by listening to his/her voice on the telephone, rather than directly asking. The reliability of interviewers’ reports of gender is unknown. Generalizability of these findings beyond the Indian Nokia user context cannot be assumed. However, given high rates of cellphone penetration in other LMICs, we expect that our findings are likely to generalize, particularly if messages are culturally tailored as they were in this study.

The results from this study demonstrate that health promotion text messaging interventions are feasible to implement in 12 languages, across a large LMIC population, and can be effective in improving behaviors that heighten the risk of diabetes and other chronic diseases. With its low cost and burden, text messaging intervention holds the potential to represent sound and effective public health investment. The cost of Arogya World’s one million-person program was $0.65 per person, including program development, transmission, and measurement. The current global epidemic of noncommunicable disease creates an urgent need for proven, simple, transportable, readily executable strategies that offer hope for chronic disease prevention at the population level particularly in LMICs. Population-level mobile health promotion interventions like the one described, show promise to meet that need. Additional evaluation using randomized trial design with measurement of objective outcomes will add to the evidence base for the use of mobile technology in chronic disease prevention.

## Appendix

Multimedia Appendix 1 mDiabetes text messages sent.

## Abbreviations

LMIC: low/middle income country
