# Supplementary material for: mHealth Intervention to Improve Diabetes Risk Behaviors in India: A Prospective, Parallel Group Cohort Study
Source: J Med Internet Res. 2016 Aug 5;18(8):e207. doi: 10.2196/jmir.5712 (PMC4992169; doi:10.2196/jmir.5712)
Supplement: Multimedia Appendix 1 [file jmir_v18i8e207_app1.pdf]

SCREENER

S1. Have you subscribed to Nokia life health channel? Please answer in Yes/ No

Yes	1	**CONTINUE**
No	2	**TERMINATE**
Don't know	3	

S2. Please tell me your age in completed years ___________________________ **(NOTE VERBATIM AND CIRCLE THE APPROPRIATE RANGE)**

Age	Code
18-24 Years	1
25-29 years	2
30-34 years	3
35-39 years	4
40-44 years	5
45-49 years	6
50-54 years	7
55-59 years	8
60-64 years	9
65 years and above	10

S3. Would you like to receive information related to diabetes by SMS? **SINGLE CODING**

Yes	1
May be/ Not Sure	2
No	3

NOT FOR FIELD STAFF. FOR DP & RESEARCH STAFF ONLY

S4. Could you please tell me name of city / state you belong to?

**City / Village**	
**State**	

S5. BASIS NAME OF CITY / STATE MENTIONED BY RESPONDENT IN S9, **INTERVIEWER TO CLASSIFY** IT AS **URBAN/RURAL** AND **NORTH / SOUTH** INDIA AND CODE BELOW

Urban	1	**CONTINUE INTERVIEWER TO MAINTAIN EQUAL QUOTA FOR BOTH URBAN / RURAL**
Rural	2	

North India	1	**INTERVIEWER TO MAINTAIN EQUAL QUOTAS FOR NORTH AND SOUTH INDIA**
South India	2	

MAIN QUESTIONNAIRE

SECTION I
AWARENESS

Now, I will ask you few questions about your general awareness about different diseases and from where you receive this information.

MQ1. Are you aware of diabetes?

Yes…………………..1 **CONTINUE**	No……………………….2 **TERMINATE**

MQ2. What causes diabetes? **DO NOT READ OUT THE OPTIONS, (MULTIPLE CODING POSSIBLE) IF RESPONDENT GIVES SOME OTHER RESPONSE THEN NOTE IN "ANY OTHER"**

Overweight/Obesity	1
Hyper tension	2
Poor eating habits	3
Lack of regular exercise	4
Inactive lifestyle	5
Family history of diabetes (From one of the parents/grandparents if they have diabetes)	6
Don't know	7
Increasing waist size	8
Others (Specify) ______________________________________	9
Others (Specify)_____________________________________	10
Others (Specify)_____________________________________	11
Others (Specify) ______________________________________	12
Others (Specify)_____________________________________	13

MQ3.What in your opinion is one of the most common complications if diabetes is left uncontrolled (**CIRCLE FIRST COMPLICATION AS TOLD BY RESPONDENT**)

MQ3a) I will now read out a few complications believed to be associated with diabetes. Please tell me if the one I am reading in your opinion is associated with diabetes?
(READ ONE BY ONE FROM TABLE BELOW. MULTIPLE CODING POSSIBLE)

**CONDITIONS**	**Spontaneous MQ 5**	**Probed MQ5a**
Loss of Vision / Cataract	1	2
Kidney Disease	1	2
Feet diseases	1	2
Heart Failure/Stroke	1	2
Poor Wound healing / Amputation	1	2
Nerve damage	1	2
Gum diseases	1	2
Others (SPECIFY) _____________________________________	1	2
Others (SPECIFY) _____________________________________	1	2
Others (SPECIFY) _____________________________________	1	2
Don't know/can't say	99	99

SECTION II
ATTITUDES AND PERCEPTION ASSOCIATED WITH DIABETES

MQ4. Different people have different views about diabetes and its management. I will read out some statements and would like to know if you agree or disagree with the statements on a scale of 1 to 5 where 5 is completely agree and 1 is completely disagree. **READ OUT THE STATEMENTS**

Statements	Agree	Disagree	Neither agree nor disagree/don't know
Children of diabetics are at higher risk of getting the disease	1	2	3
Diabetes can be cured	1	2	3
Diabetes has no effect on kidneys	1	2	3
Smoking and Alcohol intake increases risk of diabetes	1	2	3
Fasting blood sugar levels of 210 is very high	1	2	3
Life style changes is the best way to control diabetes	1	2	3
Insulin is the best way to manage diabetes	1	2	3
Being physically active reduces risk of diabetes.	1	2	3
Obesity increases risk of diabetes.	1	2	3
Regular blood glucose monitoring is important for better diabetes management	1	2	3

SECTION III
PRACTICE- LIVING WITH DIABETES

MQ5. Can you tell me if you or/and any of your family members has been diagnosed or currently have diabetes?

Response	Code	Instructions
Yes I have diabetes	1	Continue
Yes, my family member has diabetes	2	Continue
I am at risk of having diabetes because my parents/grandparent have/had diabetes	3	Continue
Yes, both me and someone in the family has diabetes	4	Continue
No neither me nor anyone in the family has diabetes	5	Go to MQ11

MQ6. Do you smoke?

Yes	1
No	2

<u>IF CODED '1' IN MQ6. ASK MQ 7 & MQ8.</u>

MQ7. How frequently do you smoke in a week?

Daily	1
5-6 Days	2
3-4 Days	3
1-2 Days	4
Less than once a week	5

MQ8. How many cigarettes/ bidis do you smoke on any given day?

More than 10	1
5-10	2
Less than 5	3

SECTION IV- PRACTICE

ASK ALL

Now I would like to ask you a few questions related to your general health practices.
MQ9. Are you careful about what you eat? WOULD YOU SAY ……**READ OUT THE OPTIONS**

Yes, always	1
Very often	2
Sometimes	4
Rarely	5
Never	6

MQ10 In the last 2 weeks, how often did you eat out in a restaurant/hotel/from street side vendor? **SINGLE CODING**

Once	1
Twice	2
Thrice	3
4-6 times	4
More than 6 times	5
Never	6

MQ 11

	Do you exercise currently?	Yes **(GO TO Q 16)**	No
**TO BE ASKED IF 'NO' ABOVE**	Are you considering beginning to exercise or any physical activity in next 30 days?	Yes	No
**TO BE ASKED IF 'NO' ABOVE**	Are you considering beginning to exercise or any physical activity in next 30 days?	Yes	No

MQ 12

	How many servings of fruits you take every day?	0-1	2-3	4 or more
**IF 0-1 SERVINGS ABOVE**	Are you planning to increase your fruits intake in next 30 days?	Yes	No	
**IF 'NO' ABOVE**	Planning to increase your fruits intake in next 60 days?	Yes	No	
	How many servings of green vegetables you take every day?	0-1	2-3	4 or more
**IF 0-1 SERVINGS ABOVE**	Are you planning to increase your green vegetables intake in next 30 days?	Yes	No	
	How many servings of green vegetables you take every day?	0-1	2-3	4 or more
**IF 0-1 SERVINGS ABOVE**	Are you planning to increase your green vegetables intake in next 30 days?	Yes	No	

MQ13.

	Do you consistently avoid eating high fat food like samosas, vadai, bajji, bondas etc.	Yes	No
**ASK IF NO ABOVE**	Do you plan to do so in next 30 days?	Yes	No
**ASK IF NO ABOVE**	How about in next 60 days?	Yes	No

MQ14a. Now, I will read out food items. You have to tell me how often in last one week, you have eaten this item (**READ ITEMS ONE BY ONE**). You may tell me the frequency in term of "Daily", "Often (more than 3 times)", "Rarely (less than 3 times)" and "Never".

Eatables	Frequency of consumption			
Chappatis/ Rotis	Daily	Often	Rarely	Never
Paranthas	Daily	Often	Rarely	Never
Idli Upma	Daily	Often	Rarely	Never
Fried Vegetables	Daily	Often	Rarely	Never
Salads	Daily	Often	Rarely	Never
Boiled Vegetables	Daily	Often	Rarely	Never
Curd	Daily	Often	Rarely	Never
Bread	Daily	Often	Rarely	Never
Milk	Daily	Often	Rarely	Never
Eggs	Daily	Often	Rarely	Never
Fish	Daily	Often	Rarely	Never
Breakfast cereals(cornflakes/ oatmeal)	Daily	Often	Rarely	Never
Meat (other than chicken)	Daily	Often	Rarely	Never
Chicken	Daily	Often	Rarely	Never
**MQ 14b.** Now, I will read names of few snacking items, do let me know in last one week how often did you have each of the item?				
Alcohol	Daily	Often	Rarely	Never
Sweets	Daily	Often	Rarely	Never
Sweetened Beverages	Daily	Often	Rarely	Never
Snacks/fast food	Daily	Often	Rarely	Never
Vadai, Dosas, Bondas	Daily	Often	Rarely	Never

MQ15. Below are few Food choices. Please tell me which one would you prefer over the other.
SINGLE CODING (READ EACH OPTION ONE BY ONE AND ASK RESPONDENT WHICH OPTION BEST IMPLIES TO HIM/HER)

a	Cola Drinks	1	Lemonade	2
b	Fruit juice	1	Whole fruit juice	2
c	Fruit Chaat	1	Aloo Tikki	2
d	Vegetable Poha	1	Samosa	2
e	Chole Bhature	1	Rajma Rice	2

MQ16. When attending a public function (marriage, parties, meetings etc), you tend to________________
SINGLE CODING. (READ EACH OPTION ONE BY ONE AND ASK RESPONDENT WHICH OPTION BEST IMPLIES TO HIM/HER)

Eat everything	1
Taste a little bit of everything	2
Eat everything other than sweets	3
I am very particular about what I eat	4

Eat only healthy options	5
Control what you eat	6

MQ17 In last one month, how often would you have__________________
<u>SINGLE CODING. (FOR EACH STATEMENT READ THE OPTIONS AVAILABLE AND ASK RESPONDENT WHICH OPTION BEST IMPLIES TO HIM/HER)</u>

	**Daily**	**Alternate days**	**Selective Days/ Weekends**	**Never**
Done any form of exercise (Yoga//Running/Jogging/In Gym/Aerobics etc.)	1	2	3	4
Played any outdoor sports like basketball, football, cricket, swimming etc.	1	2	3	4
Walking	1	2	3	4

MQ18. How often do you think you are stressed in your daily life, would you say….. READ OUT THE OPTIONS….?

Very Often	1
Quite Often	2
Rarely	3

MQ19 Can you tell me how often (**<u>READ STATEMENT ONE BY ONE FROM TABLE</u>**) ________________________
<u>SINGLE CODING (READ EACH OPTION ONE BY ONE AND ASK RESPONDENT WHICH OPTION BEST IMPLIES TO HIM/HER. INTERVIEWER TO TELL RESPONDENT THE OPTIONS GIVEN ON TOP ROW)</u>

	**Regularly do it**	**Just started doing it**	**Plan to do it; haven't started yet**	**Don't do it at all**
You consciously tend to take stairs instead of using lifts and escalators	1	2	3	4
You prefer to walk down small distances for daily chores	1	2	3	4
You tend to take short walking breaks when working in office/home	1	2	3	4
When at home, you do/help with household chores	1	2	3	4

MQ20. In general, do you seek information related to avoiding/preventing/maintaining diabetes? **<u>SINGLE CODING</u>**

Yes	1	**ASK MQ37**
Sometimes	2	
No	3	

We thank you for your time and participation in the survey; before we conclude we would like to ask you two more questions that help us understand your household profile.

Supplementary Materials

mDiabetes Text Messages
6 messages week 1 then 2 times per week – 56 messages

Week 1	Message 1	Message 2	Message 3	Message 4	Message 5	Message 6
1	*Medical* Diabetes is a huge problem. At least 5 crore people in India live with diabetes. Diabfetes kills 10 lakh Indians each year. Arogya World	*Medical* People with diabetes cannot control blood sugar properly. High blood sugar for long periods of time causes harm to the body. Arogya World	*Medical* High blood sugar from diabetes can cause problems in your eyes, kidneys, heart, feet and nerves. Arogya World	*Lifestyle* Type 2 diabetes can be avoided by doing physical activity and eating healthy foods. This has been shown by medical studies. Arogya World	*Nutrition* Healthy eating means eating plenty of fresh vegetables and fruits. Commit to eating more fresh vegetables and fruits daily. Arogya World	*Fitness* You can help avoid diabetes by being physically active. Walk to the temple or shops, climb stairs or exercise daily. Arogya World
Theory	Know Impact	Know Impact	Know Impact	Become Aware	Become Aware	Commit to Change
Word count	145 (IDF, India Diabetes Map)	147 (JDRF, High Blood Sugar)	122 (ADA, Complications)	149 (ADA, Prevention)	148 (ADA, Making Healthy Food Choices)	140 (ADA, Prevention)

Week/Topic	Message 1	Message 2
2	*Lifestyle* If you have diabetes your blood sugar can go too high or too low. Keep blood sugar in the proper range (80-120 mg/dL). Eat healthy. Walk often. Arogya World	*Nutrition* Eat fruit as a snack if you are hungry between meals. Try apples, bananas, oranges, mango and papaya. Arogya World
Theory	Control Risks	Choose Alternative
Word count	168 (ADA, Checking Your Blood Glucose)	129 (ADA, Snacks)
3	*Medical* To avoid diabetes, women should have a waist size of less than 80 cms (32 inches) and men less than 90 cms (36 inches). Arogya World	*Fitness* Decide to be physically active every day. Walk as much as you can. Try to walk more than you did last time. The extra steps are good for your health! Arogya World
Theory	Become Aware	Commit to Change
Word count	143 (H. Ranjani)	172 (ADA, Physical Activity)
4	*Medical* If you have family members with diabetes you may be at risk. Reduce your risk by taking a walk a few times each week. Arogya World	*Think* By receiving these messages you are learning to improve your health. Learning about diabetes can help you live better with the disease. Arogya World
Theory	Control Risk	Become Aware
Word count	140 (ADA, Genetics of Diabetes)	156 (IDF, Self-Management Education)
5	*Nutrition* Eat fewer high fat foods. Limit namkeens, bhajjias, samosas, ladoos, jalebis, halwa. Avoid food made in ghee. Arogya World (NORTH)<br><br>*Nutrition* Eat fewer high fat foods. Limit bhajji, bondas, vadas, jhangri, badusha. Avoid food made in ghee or coconut oil. Arogya World (SOUTH)	*Fitness* Walk more each week. Week 1: walk 10 minutes on 2 days of the week. Week 2: walk 10 minutes on 3 days. Week 3: walk 15 minutes on 3 days, and so on. Arogya World
	Choose Alternatives	Commit to Change
Word count	139 (NORTH) 141 (SOUTH) (ADA, Fats and Diabetes)	172 (ADA, Food and Fitness)
6	*Nutrition*Try eating small meals and snacks throughout day to maintain blood sugar. Long gaps between meals can cause your blood sugar to drop. Arogya World	*Nutrition* Eat healthy foods rich in protein. Try low fat curds, low fat milk, beans, eggs, pulses, nuts, chicken and fish. Eat these to avoid diabetes. Arogya World
	Control Risk	Choose Alternatives
Word count	157 (ADA, Snacks)	173 (ADA, Lean Meats)
7	*Fitness* Be physically active to achieve a proper weight and healthy waist size. Being overweight and having a large waist size increase risk for diabetes. Arogya World	*Nutrition* Eat less fried foods and sweets. Take larger amounts of vegetables and fruits. This will help avoid diabetes. Arogya World
	Control Risks	Choose Alternatives
Word count	169 (ADA, Overweight)	134 (ADA, Making Healthy Food Choices)
8	*Medical* Diabetes is the most common cause of kidney failure. If you have diabetes, ask your doctor during the next visit to learn more about kidney disease. Arogya World	*Nutrition* To help avoid diabetes, eat vegetables. Try carrots, green beans, spinach, sprouts, cucumber, cauliflower, cabbage, capsicum and gourds. Arogya World
	Know Impact/Seek Support	Choose Alternatives
Word count	172 (NKUDIC, Kidney Disease of Diabetes)	170 (ADA, Non-Starchy Vegetables)
9	*Medical* If you are overweight or have a large waist size then you are at risk for diabetes. Control your weight. Arogya World	*Nutrition* Small changes in the way you eat can help you avoid diabetes. Eat more vegetables and fruits. Eat less rice and roti. Arogya World
	Control Risks	Choose Alternatives
Word count	127 (ADA, Overweight)	142 (ADA, Making Healthy Food Choices)
10	*Medical* Tobacco is harmful to health and makes diabetes complications more severe. Avoid smoking cigarettes and chewing tobacco. Arogya World	*Fitness* Do physical activity. Daily activities are walking, climbing stairs or household work. Planned exercise is a morning walk, sports or yoga. Arogya World
	Control Risks	Commit to Change
Word count	143 (Mayo Clinic, Diabetes Care)	163 (ADA, Physical Activity)
11	*Think* Do you know someone with type 2 diabetes? Tell them that healthy eating and physical activity can help them control their diabetes. Arogya World	*Medical* Medical studies show that you should do daily physical activity and eat healthy foods to help you avoid type 2 diabetes. Arogya World
	Seek Support	Know Impact
Word count	152 (NKUDIC, Kidney Disease of Diabetes)	143 (NKUDIC, Kidney Disease of Diabetes)
12	*Medical* Diabetes causes kidney disease, heart disease, eye disease, skin disease and foot ulcers. These are called diabetes complications. Arogya	*Fitness * Take a morning or evening walk to help avoid diabetes. Use footwear. Shoes made for walking help avoid injury. Arogya World

	World	
	Know Impact	Commit to Change
Word count	156 (ADA, Complications)	134 (ADA, Walk Away a Winner)
13	*Medical* If you have diabetes in your family you are at risk for getting diabetes yourself. Go to a clinic to have your blood sugar checked. Arogya World	*Lifestyle* Send an SMS to a friend to join you for a walk, join a gym or go to yoga class. Physical activity is more fun with a friend! Arogya World
	Control Risks	Seek Support
Word count	154 (ADA, Genetics of Diabetes)	150 (ADA, Physical Activity)
14	*Medical* Walking more and eating healthy foods can help protect your heart. Heart disease is responsible for 50-80% of deaths in people with diabetes. Arogya World	*Lifestyle* A proper weight and healthy waist size can help you manage diabetes. Eat less fat. Reduce sugary sweets. Be physically active often. Arogya World
	Know Impact/Become Aware	Commit to Change
Word count	165 (WHO, Diabetes)	157 (ADA, Overweight)
15	*Medical* If you have diabetes, visit an eye doctor (ophthalmologist). People with diabetes are 25 times more likely to develop blindness. Arogya World	*Fitness* Add a little physical activity each day to avoid diabetes. Climb stairs. Walk to temple, mosque, church or vegetable shop. Arogya World
	Seek Support	Commit to Change
Word count	152 (CDC,MMWR)	147 (ADA, Physical Activity)
16	*Medical* Did you know high blood sugar can increase your risk for heart disease? 50-80% of people with diabetes die of heart disease. Arogya World	*Think* To live a healthy life, learn as much as you can about diabetes. Manage diabetes by eating healthy food and walking often. Arogya World
	Know Impact	Become Aware
Word count	147 (WHO, Diabetes)	144 (WHO, Self-Management Education)
17	*Medical* Type 2 diabetes can be avoided, but once a person gets diabetes it cannot be cured. Fortunately you can avoid diabetes by living a healthy lifestyle. Arogya World	*Nutrition* Eat a healthy breakfast every day. Eating breakfast gives you energy and helps maintain your blood sugar levels properly. Arogya World
	Become Aware	Commit to Change
Word count	174 (H.Ranjani)	146 (ADA, Breakfast on the Go)
18	*Lifestyle* If you smoke, especially if you have diabetes, take steps to quit smoking today. Ask family and friends for support. Arogya World	*Medical* If you have diabetes, keep your blood sugar levels in proper range. Eat healthy foods. Being physically active. Sometimes medications are needed. Arogya World
	Control Risks/Seek Support	Control Risks
Word count	143 (Mayo Clinic, Diabetes Care)	169 (IDF, Management of Diabetes)
19	*Medical* There is a test to see if your kidneys are working properly. If you have diabetes, ask your doctor to perform this test at your next visit. Arogya World	*Nutrition* Fruits are good to eat if you are hungry between meals. Try apples, bananas, mosambi, grapes, mango, papaya, guava, sapota and jackfruit. Arogya World
	Become Aware/Seek Support	Choose Alternatives
Word count	163 (NKUDIC, Kidney Disease of Diabetes)	168 (ADA, Fruits)
20	*Medical* The highest number of deaths from diabetes happen in developing countries like India and China. Diabetes kills 10 lakh Indians each year. Arogya World	*Nutrition* Eat healthy whole grains to help avoid diabetes. Try bajra, jowar, ragi, pulses, oats, brown rice, barley and whole wheat atta. Arogya World
	Know Impact	Choose Alternatives
Word count	160 (IDF, Diabetes Atlas)	158 (ADA, Whole Grains)
21	*Medical* The World Health Organization says that eating healthy, doing physical activity and avoiding tobacco can prevent 80% of diabetes cases. Arogya World	*Think* If you have diabetes you can still live a normal life. Learn as much as you can about diabetes and maintaining your blood sugar. Arogya World
	Control Risks/Benefit	Become Aware/Benefit
Word count	178 (WHO, Unhealthy Diets & Physical Inactivity)	150 (IDF, Self-Management Education)
22	*Lifestyle* Stopping smoking is one of the best things you can do for your health! If you want to stop smoking but are having trouble, get help from your doctor. Arogya World	*Nutrition* Take larger amounts of foods like raw vegetables and fresh fruits. Take smaller amounts of sugary sweets like gulab jamoon, and jilebi. Arogya World (NORTH)<br><br>*Nutrition* Take larger amounts of foods like raw vegetables and fresh fruits. Take smaller amounts of sugary sweets like mysore pak or jilebi. Arogya World (SOUTH)<br>*Nutrition* Take larger amounts of foods like raw vegetables and fresh fruits. Take smaller amounts of

		sugary sweets like rasagulla or sandesh. Arogya World (EAST)
	Seek Support	Choose Alternatives
Word count	175 (ADA, Smoking)	161 (NORTH) 156 (SOUTH) 156 (EAST) (ADA, Making Healthy Food Choices)
23	*Lifestyle* Changing habits is difficult. Pat yourself on the back for even small changes you have made. What changes have you made to improve your health? Arogya World	*Nutrition* If you do not like the taste of raw vegetables, flavor them with different masalas or squeeze lemon juice on them. Try cucumbers, carrots or tomatoes. Arogya World
	Reward Self	Choose Alternatives
Word count	169	177 (MyPlate, Tips to help you eat vegetables)
24	*Medical* Prolonged periods of high blood sugar from diabetes can cause problems in your eyes, kidneys, heart, and feet. These are called diabetes complications. Arogya World	*Think* Half of what you eat should be vegetables like carrots, tomatoes. One quarter should be rice, chappati and the rest protein like eggs, dal. Include curds. Arogya World
	Know Impact	Choose Alternatives
Word count	178 (ADA, Hypoglycemia)	178 DCLIP (MDRF-EMORY)
25	*Medical* Stop using tobacco. If you have diabetes, smoking cigarettes and chewing tobacco makes diabetes complications (heart disease, nerve damage) more severe. Arogya World	*Medical* Manage diabetes by eating healthy foods like vegetables and fruits and doing physical activity. Arogya World
	Control Risks	Commit to Change
Word count	177 (Mayo Clinic, Diabetes Care)	118 (DPP, Overview; H. Ranjani)
26	*Think* Learn as much as you can about diabetes. This can help you take care of yourself and your family. Arogya World (FOR WOMEN)<br><br>*Think* Learn as much as you can about diabetes. Knowing how to care for yourself can help you stay healthy and active. Arogya World (FOR MEN)	*Fitness* Be physically active every day. Go for a walk. Physical activity can help lower your risk for heart disease and diabetes. Arogya World
	Become Aware	Control Risks
Word count	120 (WOMEN) 133 (MEN)	145 (ADA, Physical Activity)
